# Supplementary material for: Fine Mapping of Glutamate Decarboxylase 65 Epitopes Reveals Dependency on Hydrophobic Amino Acids for Specific Interactions
Source: Int J Mol Sci. 2019 Jun 14;20(12):2909. doi: 10.3390/ijms20122909 (PMC6627456; doi:10.3390/ijms20122909)
Supplement: Supplementary file 1 [file ijms-20-02909-s001.pdf]

Scheme 1. Peptides used for epitope identification and characterization.

>sp|Q05329.1| Full65 kDa glutamic acid decarboxylase; Short=GAD65

<u>0–60</u>

MASPGSGFWS FGSEDGSGDS ENPGTARAWC QVAQKFTGGI GNKLCALLYG

MASPGSGFWS FGSEDGSGDS (1)

FGSEDGSGDS ENPGTARAWC (2)

ENPGTARAWC QVAQKFTGGI (3)

QVAQKFTGGI GNKLCALLYG

<u>351–400</u>

ADICKKYKIW MHVDAAWGGG LLMSRKHKWK LSGVERANSV TWNPHKMMGV

ADICKKYKIW MHVDAAWGGG (36)

MHVDAAWGGG LLMSRKHKWK (37)

LLMSRKHKWK LSGVERANSV (38)

LSGVERANSV TWNPHKMMGV (39)

TWNPHKMMGV PLQCSALLVR (40)

<u>401–450</u>

PLQCSALLVR EEGLMQNCNQ MHASYLFQQD KHYDLSYDTG DKALQCGRHV

PLQCSALLVR EEGLMQNCNQ (41)

EEGLMQNCNQ MHASYLFQQD (42)

MHASYLFQQD KHYDLSYDTG (43)

KHYDLSYDTG DKALQCGRHV (44)

DKALQCGRHV DVFKLWLMWR (45)

<u>451–500</u>

DVFKLWLMWR AKGTTGFEAH VDKCLELAEY LYNIIKNREG YEMVFDGKPQ

DVFKLWLMWR AKGTTGFEAH (46)

AKGTTGFEAH VDKCLELAEY (47)

VDKCLELAEY LYNIIKNREG (48)

LYNIIKNREG YEMVFDGKPQ (49)

YEMVFDGKPQ HTNVCFWYIP (50)

<u>501–550</u>

HTNVCFWYIP PSLRTLEDNE ERMSRLSKVA PVIKARMMEY GTTMVSYQPL

HTNVCFWYIP PSLRTLEDNE (51)

PSLRTLEDNE ERMSRLSKVA (52)

ERMSRLSKVA PVIKARMMEY (53)

PVIKARMMEY GTTMVSYQPL (54)

GTTMVSYQPL GDKVNFFRMV (55)

<u>551–585</u>

GDKVNFFRMV ISNPAATHQD IDFLIEEIER LGQDL

GDKVNFFRMV ISNPAATHQD (56)

ISNPAATHQD IDFLIEEIER (57)

IDFLIEEIER LGQDL (58)

Terminally truncated resin-bound peptides for identification of N- and C-terminal borders

pAb [2] epitope 1:

PSLRT	RLSKV
PSLRTL	SRLSKV
PSLRTLE	MSRLSKV
PSLRTLED	RMSRLSKV
PSLRTLEDN	ERMSRLSKV
PSLRTLEDNE	EERMSRLSKV
PSLRTLEDNEE	NEERMSRLSKV
PSLRTLEDNEER	DNEERMSRLSKV
PSLRTLEDNEERM	EDNEERMSRLSKV
PSLRTLEDNEERMS	LEDNEERMSRLSKV
PSLRTLEDNEERMSR	TLEDNEERMSRLSKV
PSLRTLEDNEERMSRL	RTLEDNEERMSRLSKV
PSLRTLEDNEERMSRLS	LRTLEDNEERMSRLSKV
PSLRTLEDNEERMSRLSK	SLRTLEDNEERMSRLSKV
PSLRTLEDNEERMSRLSKV	PSLRTLEDNEERMSRLSKV

pAb [2] epitope 2:

MVSY	VNFF
MVSYQ	KVNFF
MVSYQP	DKVNFF
MVSYQPL	GDKVNFF
MVSYQPLG	LGDKVNFF
MVSYQPLGD	PLGDKVNFF
MVSYQPLGDK	QPLGDKVNFF
MVSYQPLGDKV	YQPLGDKVNFF
MVSYQPLGDKVN	SYQPLGDKVNFF
MVSYQPLGDKVNF	VSYQPLGDKVNFF
MVSYQPLGDKVNFF	MVSYQPLGDKVNFF

mAb [144]:

GSGF	DGSG
GSGFW	EDGSG
GSGFWS	SEDGSG
GSGFWSF	GSEDGSG
GSGFWSFG	FGSEDGSG
GSGFWSFGS	SFGSEDGSG
GSGFWSFGSE	WSFGSEDGSG
GSGFWSFGSED	FWSFGSEDGSG
GSGFWSFGSEDG	GFWSFGSEDGSG
GSGFWSFGSEDGS	SGFWSFGSEDGSG
GSGFWSFGSEDGSG	GSGFWSFGSEDGSG
PGSGFWSFGSEDGSGDSEN	PGSGFWSFGSEDGSGDSEN

Free peptides for identification of complete epitope

mAb [144]:

(truncated)	(Alanine-substituted)	(Functionality-substituted)
FWSFGSE	AWSFGSE	WWSFGSE
GFWSFGSE	FASFGSE	FFSFGSE
GFWSFGSED	FWAFGSE	FWTFGSE
SGFWSFGSED	FWSAGSE	FWSWGSE
SGFWSFGSEDG	FWSFASE	FWSFGTE
PGSGFWSFGSEDGSGDSEN	FWSFGAE	FWSFGSD
	FWSFGSA	

pAb [2] epitope 1:

(truncated)	(Alanine-substituted)	(Functionality-substituted)
TLED	ATLED	KTLED
RTLED	RALED	RSLED
RTLEDN	RTAED	RTIED
LRTLEDN	RTLAD	RTLDD
LRTLEDNE	RTLEA	RTLEE
SLRTLEDNE		
SLRTLEDNEE		
PSLRTLEDNEERMSRLSK		

pAb [2] epitope 2:

(truncated)	(Alanine-substituted)	(Functionality-substituted)
PLGDKVN	ALGDKVNF	PIGDKVNF
PLGDKVNF	PAGDKVNF	PLGEKVNF
YQPLGDKVNF	PLADKVNF	PLGDRVNF
YQPLGDKVNFF	PLGAKVNF	PLGDKLNF
YQPLGDKVNFFR	PLGDAVNF	PLGDKVQF
TTMVSYQPLGDKVNFFRM	PLGDKANF	
	PLGDKVAF	
	PLGDKVNF	
